# Male Breast Cancer: Epidemiology, Diagnosis, Molecular Mechanisms, Therapeutics, and Future Prospective

**DOI:** 10.32604/or.2025.068238

**Published:** 2025-12-30

**Authors:** Ashok Kumar Sah, Ranjay Kumar Choudhary, Velilyaeva Alie Sabrievna, Karomatov Inomdzhon Dzhuraevich, Anass M. Abbas, Manar G. Shalabi, Nadeem Ahmad Siddique, Raji Rubayyi Alshammari, Navjyot Trivedi, Rabab H. Elshaikh

**Affiliations:** 1 Department of Medical Laboratory Sciences, College of Applied & Health Sciences, A Sharqiyah University, Ibra, 400, Oman; 2 School of Allied Health Sciences, Sanskaram University, Jhajjar, 124108, Haryana, India; 3 Department of Psychiatry, Medical Psychology and Narcology, Samarkand State Medical University, Samarqand, 140100, Republic of Uzbekistan; 4 Department of Folk Medicine and Professional Diseases, Bukhara State Medical Institute, Bukhara, 200118, Republic of Uzbekistan; 5 Department of Clinical Laboratory Sciences, College of Applied Medical Sciences, Jouf University, Sakaka, 72388, Saudi Arabia; 6 Department of Pharmaceutical Chemistry, University of Hafar Al Batin, Hafar Al Batin, 31991, Saudi Arabia; 7 Department of Pharmacy Practice, University of Hafar Al Batin, Hafar Al Batin, 31991, Saudi Arabia; 8 Department of Physiotherapy, University Institute of Allied Health Sciences, Chandigarh University, Mohali, 140413, Punjab, India

**Keywords:** Male breast cancer, epidemiology, diagnostic strategies, molecular profiling, therapeutic advances, precision oncology, prognostic biomarkers, multiomics, personalized medicine

## Abstract

Male breast cancer (MBC) is rare, representing 0.5%–1% of all breast cancers, but its incidence is increasing due to improved diagnostics and awareness. MBC typically presents in older men, is human epidermal growth factor receptor 2 (HER2)-negative and estrogen receptor (ER)-positive, and lacks routine screening, leading to delayed diagnosis and advanced disease. Major risk factors include hormonal imbalance, radiation exposure, obesity, alcohol use, and Breast Cancer Gene 1 and 2 (BRCA1/2) mutations. Clinically, it may resemble gynecomastia but usually appears as a unilateral, painless mass or nipple discharge. Advances in imaging and liquid biopsy have enhanced early detection. Molecular mechanisms involve hormonal signaling, HER2/epidermal growth factor receptor (EGFR) pathways, tumor suppressor gene alterations, and epigenetic changes. While standard treatments mirror those for female breast cancer, emerging options such as cyclin-dependent kinase 4 and 6 (CDK4/6), and poly(ADP-ribose) polymerase (PARP) inhibitors, immunotherapy, and precision medicine are reshaping management. Incorporating artificial intelligence, molecular profiling, and male-specific clinical trials is essential to improve outcomes and bridge current diagnostic and therapeutic gaps.

## 1 Introduction

Male breast cancer (MBC) is rare, comprising approximately 0.5%–1% of all breast cancer cases worldwide, translating to about 1 in 100 diagnosed breast cancer cases [1]. Global epidemiological trends have revealed a steady increase in the incidence of MBC over the past two decades, likely due to improved diagnostic capabilities and greater awareness. Data derived from the Surveillance, Epidemiology, and End Results (SEER) program showed an annual increase in MBC incidence rates of 1.26% from 1975 to 2017, underscoring the need for targeted public health interventions [2]. The prevalence of MBC varies significantly across regions, with higher rates reported in North America and Europe than in Asia and Africa, reflecting geographic, genetic, and environmental differences [3].

A comparison of MBC and female breast cancer (FBC) highlights distinct differences in clinical presentation, biology, and outcomes. MBC patients are usually diagnosed later, with a mean age of 65–70 years, compared to 55–60 years for FBC [4]. Unlike FBC, where mammography facilitates early detection, the lack of routine [5]. Furthermore, MBC tends to exhibit a higher proportion of estrogen receptor (ER)-positive and human epidermal growth factor receptor 2 (HER2)-negative tumors, which have implications for therapeutic strategies [6]. These differences emphasize the importance of distinct clinical management protocols tailored to the unique characteristics of MBC. Socioeconomic and healthcare disparities convoluted the challenges associated with MBC. Studies have shown that men with low socioeconomic backgrounds and underserved communities face significant barriers to timely diagnosis and treatment. For example, racial and ethnic disparities are evident in the United States, where African-American men with MBC are more likely to present with advanced-stage disease and have worse overall survival than their Caucasian counterparts [7]. These disparities are attributed to factors such as limited access to healthcare facilities, underrepresentation in clinical trials, and lack of awareness among both patients and healthcare providers [8]. Globally, similar trends are observed in low- and middle-income countries, where inadequate healthcare infrastructure and cultural stigmas further hinder early diagnosis and management [9].

Understanding the potential of precision medicine in MBC management will also be provided by a thorough examination of new diagnostic technologies and individual treatment strategies. Furthermore, our knowledge of MBC pathogenesis has changed because of advances in molecular biology, opening the door to new treatment options. In doing so, this review emphasizes how crucial it is to use a multidisciplinary approach to handle the intricacies of this uncommon but significant illness.

Here, we aim to close this gap by offering a thorough and targeted analysis of the combination of artificial intelligence (AI) and precision oncology within the MBC framework. In contrast to earlier reviews that mostly focused on traditional clinical viewpoints, we emphasize how AI-driven technologies, including radiomics, machine learning algorithms, and genomics-based predictive models, have the potential to completely transform prognostic evaluation, tailored treatment planning, and early diagnosis, particularly in MBC patients. The function of multiomics techniques and the possibilities of personalized therapy strategies catered to the unique molecular features of MBC were also investigated. By highlighting how AI technologies and precision oncology cross in MBC, we provide a fresh perspective and want to spur more research to define this important field of oncology.

By applying keywords associated with MBC, we conducted a systematic search of PubMed, Web of Science, and Scopus until January 2025. These keywords included “breast cancer”, “male breast cancer”, “incidence”, “deaths”, “prevalence”, “disability-adjusted life-years”, “global burden”, “epidemiology”, “treatment”, “management”, “advances in treatment”, and “trend analysis”. Due to MBC’s rarity, there has not been much research done on it globally, and the majority of studies have been region-specific or based on out-of-date data. Insufficient knowledge is available regarding how MBC affects the total cancer burden, especially in relation to risk factors, trends over time, and advancements in AI-assisted treatment.

## 2 Epidemiology and Risk Factors of Male Breast Cancer

The distribution of MBC varies significantly with age and location, reflecting the interaction of lifestyle, environmental, and genetic factors. One distinctive demographic feature of MBC is that its patients have a median age at diagnosis of approximately 67 years, which is higher than that of FBC patients [10]. About 38,827 new cases, 320,459 prevalent cases, 13,274 deaths, and 380,917 disability-adjusted life years (DALYs) were reported worldwide in 2023, and the largest disease burden was found in Eastern Sub-Saharan Africa. The age-standardized incidence and death rates of MBC have increased dramatically between 1990 and 2021 [3]. Nonetheless, forecasts suggest that these rates may decrease over the next three decades.

The Eastern Sub-Saharan Africa region had the highest age-standardized rates of incidence (3.05%), prevalence (17.86%), mortality (2.39%), and DALYs (57.76%). In contrast, the high-middle Socio-demographic Index (SDI) region showed the highest incidence (1.16%) and prevalence (10.22%) rates, while the low SDI region reported the highest mortality (0.89%) and DALY (21.71, 95% Uncertainty Interval (UI): 16.08–34.41) rates [3,11]. Among individual countries, Uganda exhibited the highest age-standardized rates for all key metrics: incidence (4.54%), prevalence (26.28%), mortality (3.51%), and DALYs (84.59%). Globally, the peak incidence (6,492 cases), prevalence (53,543 cases), and mortality (2,018 deaths) were observed in the 65–69-year age group. The highest DALY burden (54,363 DALYs) was observed among individuals aged 60–64 years. Age-specific analyses revealed a gradual increase in the incidence, prevalence, mortality, and DALYs with advancing age. The highest age-specific rates were noted in individuals aged ≥ 95 years for DALYs (55.73%) and mortality (6.56%), whereas those aged 85–89 years exhibited the highest prevalence (49.23%) and incidence (6.76%) [3,11].

From 1990 to 2023, East Asia showed the highest increase in age-standardized incidence (4.50%) and prevalence (4.61%) of MBC, while Tropical Latin America recorded the largest rise in mortality and DALYs (Average Annual Percentage Change (AAPC): 2.02% each). In contrast, Eastern Europe exhibited the most significant decline in mortality and DALY rates. The middle SDI region experienced the greatest rise across all metrics—incidence, prevalence, deaths, and DALYs—while high SDI and low SDI regions showed notable declines in deaths and DALYs, respectively. Georgia showed the sharpest increases in incidence (25.44%), prevalence (7.43%), mortality (AAPC: 25.08%), and DALYs (AAPC: 20.17%). Conversely, Belarus showed the most substantial decrease, with an AAPC of −8.14% for incidence, −3.14% for prevalence, −9.54% for mortality, and −9.45% for DALYs [3,11].

Age-specific trends revealed a significant increase in MBC risk after the age of 60 years, coinciding with changes in hormonal levels and cumulative environmental exposures. Notably, a subset of cases has been identified in younger men, often linked to hereditary factors such as BRCA2 mutations, underscoring the role of genetic predisposition in the early onset of MBC [12–14]. Familial clustering occurs in 15%–20% of MBC cases and is frequently linked to germline mutations in the *BRCA1*, *BRCA2*, and *CHEK2* genes. *BRCA2* mutations are particularly significant, as they confer a lifetime risk of breast cancer of up to 8% in men, which is markedly higher than the general male population risk of 0.1% [12–14]. Moreover, emerging studies have identified additional genetic risk factors, such as mutations in *PALB2* and *PTEN*, thus expanding our understanding of hereditary components in MBC [14,15]. Hormonal dysregulation is another critical determinant of MBC pathogenesis. Elevated estrogen levels, often secondary to conditions such as obesity, liver cirrhosis, or exogenous hormone exposure, contribute to an increased risk. Additionally, androgen deficiency, including hypogonadism and Klinefelter syndrome, significantly increases the risk of MBC [16]. Klinefelter syndrome, characterized by the presence of an extra X chromosome, has been associated with a 20–50 times higher risk of MBC compared to the general male population, highlighting the importance of hormonal influences [17].

The risk of MBC is also significantly influenced by lifestyle factors. Because androgens in adipose tissue aromatize peripherally, obesity, a known risk factor, is associated with higher levels of circulating estrogens [18]. Research by Guénel et al. has indicated a dose-response association between alcohol intake and illness development, and chronic alcohol use has been associated with elevated estrogen levels and consequent MBC risk [18]. Moreover, exposure to ionizing radiation, particularly during childhood or adolescence, has been implicated in the development of MBC later in life. Environmental pollutants, such as endocrine-disrupting chemicals, are also gaining attention as potential contributors to disease etiology, necessitating further research [19].

The distinction between familial and sporadic MBC is critical for understanding its epidemiology. Although familial cases are predominantly associated with genetic mutations, sporadic cases often result from a complex interplay between hormonal, environmental, and lifestyle factors [20]. Such as, sporadic MBC has been linked to occupational exposure, such as in men working in high-temperature environments or those exposed to exhaust fumes and other carcinogens [21]. This underscores the multifactorial nature of MBC and the need for comprehensive risk assessment models. Recent epidemiological studies have shed light on the global and regional burden of MBC. In the United States, data from the National Cancer Database indicate an annual increase in incidence, reflecting better awareness and diagnostic practices [22]. Similar trends have been reported in European countries, where population-based cancer registries have documented a gradual increase in cases over the last two decades [23]. However, in low- and middle-income countries, limited data availability poses challenges in understanding disease prevalence and outcomes. Collaborative efforts are needed to establish robust cancer registries and conduct large-scale studies to fill these knowledge gaps.

## 3 Clinical Presentation and Diagnosis

MBC commonly presents as a painless unilateral lump in the breast tissue, often located beneath the nipple. This initial symptom is reported in approximately 85%–90% of the cases, underscoring its significance as a hallmark presentation [24,25]. Additional symptoms include nipple discharge, which may be serous or bloody, and observed in approximately 30% of the patients, as well as retraction, ulceration, or scaling of the nipple [24,25]. Skin changes, such as erythema, dimpling, or peau d’orange, are also common, particularly in more advanced stages. However, benign gynecomastia can complicate clinical evaluation, often leading to misdiagnosis or delays in medical attention [26].

The challenges in diagnosing MBC stem from its rarity and the general lack of awareness among both patients and healthcare providers. Unlike FBC, where routine screening programs detect many asymptomatic cases, MBC is often diagnosed at a more advanced stage, with tumors larger than 2 cm and evidence of lymph node (LN) involvement in 40%–50% of cases [22]. Cultural stigmas, reluctance among men to report breast symptoms, and misattribution of lumps to gynecomastia or trauma further delay timely diagnosis. These factors contribute to worse survival outcomes in MBC as compared to FBC, highlighting the need for heightened awareness and early diagnostic interventions [27].

Clinical examination and diagnostic approaches for MBC include clinical evaluation, imaging, pathology, and molecular profiling. Clinical examination typically identifies a firm, immobile mass beneath the nipple or areola, which is often associated with skin or nipple changes. Physical assessment of the contralateral breast and axillary lymph nodes (ALNs) is critical for detecting bilateral disease or regional metastasis [28]. Given that many cases present at advanced stages, a thorough systemic examination is recommended to rule out distant metastases.

Imaging modalities are critical for the evaluation of MBC. Mammography, the primary imaging modality, reveals characteristic findings such as eccentric masses with irregular margins or microcalcifications in nearly 90% of cases. However, its sensitivity may be reduced in dense male breast tissues. Ultrasound serves as a complementary tool, providing superior delineation of tumor margins and detection of axillary lymphadenopathy [29]. Magnetic resonance imaging (MRI) offers enhanced sensitivity in cases with equivocal findings on mammography or ultrasound and is particularly useful for detecting multifocal disease or local recurrence [29]. Despite its strengths, MRI’s high cost and limited availability restrict its routine use in MBC.

Pathological examination remains the gold standard for the diagnosis of MBC. Core needle biopsy, which is preferred over fine-needle aspiration cytology (FNAC), provides sufficient tissue for histopathological evaluation and biomarker analysis. Although less invasive, FNAC may yield inadequate samples, particularly in cases of fibrotic or necrotic tumors [30]. Histologically, invasive ductal carcinoma accounts for over 90% of MBC cases, with other subtypes, such as invasive lobular carcinoma, being exceedingly rare due to the paucity of lobular structures in the male breast [30].

Immunohistochemistry (IHC) is essential for assessing hormone receptor status and guiding treatment decisions. The majority of MBC cases are positive for estrogen receptors (ER, 80%–90%), progesterone receptors (PR, 65%–75%), and HER2-negative, a profile distinct from that seen in FBC [31]. Ki-67, a proliferation marker, is routinely evaluated to determine tumor aggressiveness. Emerging biomarkers, including the androgen receptor (AR) and programmed death-ligand 1 (PD-L1), are being investigated for their prognostic and therapeutic implications [32].

Emerging diagnostic tools are transforming the MBC evaluation landscape. Liquid biopsy, which involves the analysis of circulating tumor DNA (ctDNA) or circulating tumor cells (CTCs) in the bloodstream, is a minimally invasive method for early detection, monitoring of treatment response, and identification of molecular alterations [33]. Advances in genetic profiling using next-generation sequencing (NGS) have enabled the identification of somatic mutations and genomic aberrations unique to MBC, thus paving the way for precision oncology [34].

A comparative analysis with FBC revealed notable differences in the diagnostic protocols. Although mammography is effective for both sexes, its diagnostic sensitivity is often reduced in men due to the lack of routine screening and the dense nature of male breast tissue [29]. Additionally, the predominance of ER-positive tumors in MBC necessitates greater emphasis on hormone receptor analysis, whereas HER2-targeted therapies play a more significant role in FBC management [29]. These distinctions highlight the need for gender-specific diagnostic algorithms and therapeutic strategies to optimize outcomes in MBC patients.

MBC is a rare disease, and despite its rarity, MBC is associated with poorer survival outcomes than FBC. This rarity has led to limited research and awareness, resulting in disparities in clinical outcomes between MBC and FBC [29].

### 3.1 Clinical Outcomes

#### 3.1.1 Survival Rates

Investigation has indicated that the overall survival rate for men with breast cancer is sometimes lower than that of women. According to research, men’s 5-year overall survival was 77.6%, while women’s was 86.4%. Furthermore, studies show that the death rate for MBC patients is 19% higher than that of female patients [35,36].

#### 3.1.2 Stage at Diagnosis

Usually, men receive a diagnosis when the illness is further advanced. This delay, which results in later discovery and consequently worse prognoses, is frequently caused by ignorance and a lack of systematic screening [35].

#### 3.1.3 Treatment Disparities

Treatment regimens for males are frequently derived from data for women because there are few clinical studies specifically designed for men. This method might not take gender-specific biological characteristics into consideration, which could have an impact on treatment results and efficacy. Additionally, routine therapies like endocrine therapy, which can affect survival chances, are less likely to be administered to men [37].

#### 3.1.4 Socioeconomic and Lifestyle Factors

Cancer risk and outcome can be influenced by factors including obesity, smoking, and alcohol use, which are more common in some populations. According to one study, FBC survivors in underprivileged areas were 35% more likely to get secondary unrelated malignancies, indicating that socioeconomic variables influence the prognosis of cancer [38].

A comprehensive strategy is necessary to enhance MBC’s clinical results. Since MBC is a rare but serious health issue, increasing public and healthcare professional awareness is essential to encouraging earlier discovery and diagnosis. Education campaigns should emphasize the symptoms and risk factors unique to men. To provide gender-specific treatment guidelines, more funding for MBC-focused research is also essential, including the inclusion of men in clinical trials. This cause is presently being advanced by groups like the MBC Global Alliance [39]. Furthermore, early detection can be further facilitated by creating tailored screening techniques for high-risk populations, such as those with a strong family history or BRCA mutations. Thus, it is advised to encourage routine self-examinations and clinical observation in these groups. Lastly, customized psychosocial support, such as counseling and support groups, can greatly improve quality of life and lessen stigma. By addressing these important issues, men with breast cancer will have better outcomes, and the care gap will be reduced [40].

## 4 Molecular Mechanisms Underpinning MBC

**Figure 1:**
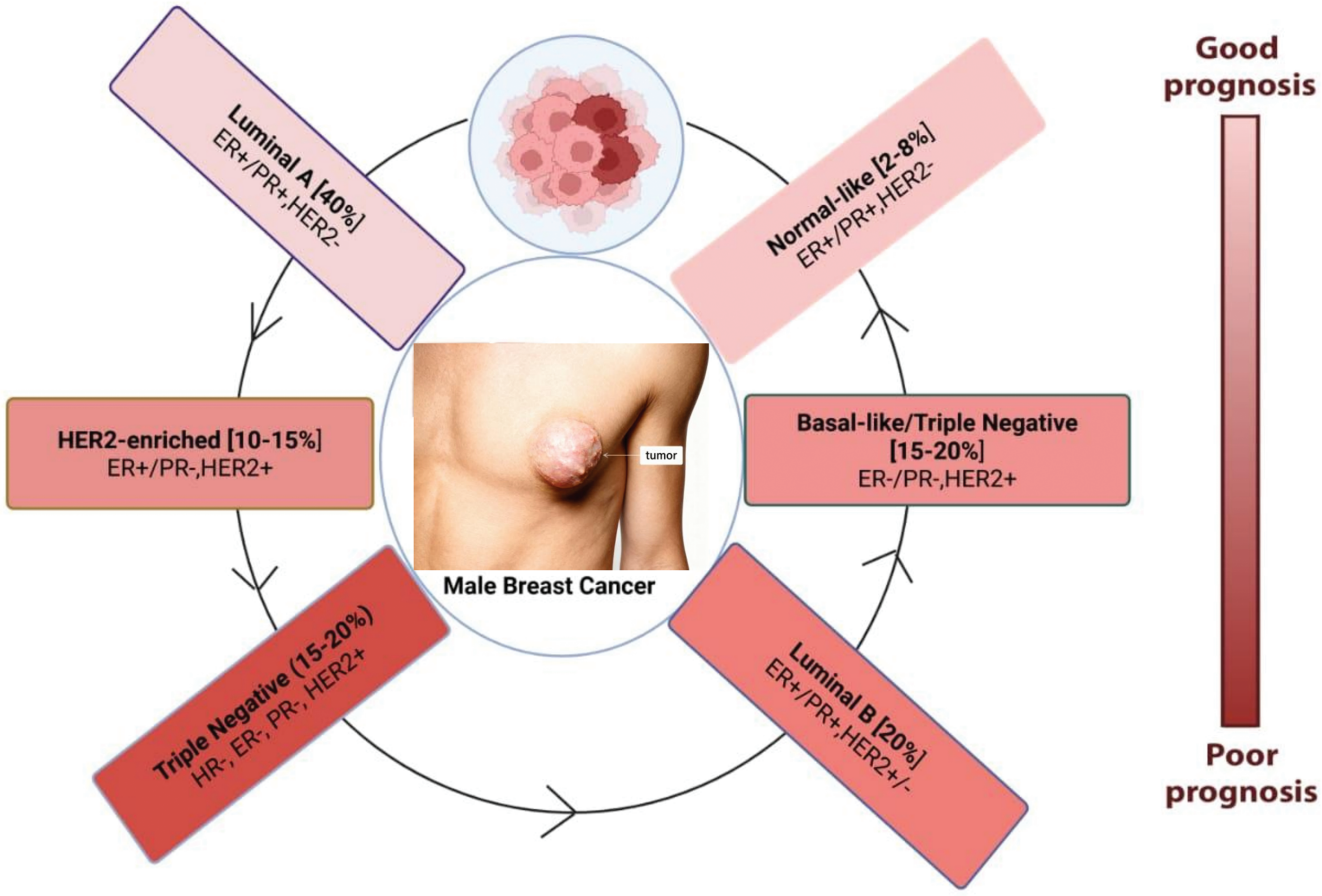
Molecular subtypes of male breast cancer and associated prognosis

The evolution of MBC is controlled by intricate molecular pathways, of which hormone signaling is a key component. More than 85% of MBC cases had activated ER and PR pathways, highlighting the reliance of tumor growth on hormonal stimulation [41]. Through both genetic and non-genomic pathways, abnormal cell proliferation is fueled by elevated estrogen levels, which are frequently brought on by obesity or diseases like Klinefelter syndrome. MBC has been linked to the androgen receptor (AR) axis, which has historically been linked to prostate cancer. Research suggests that AR expressions in ER-negative tumors may be a prognostic indicator and possible therapeutic target [42]. Different subtypes of MBC have been shown in Fig. 1.

Another important mechanism in MBC is HER2/EGFR signaling, albeit men are less likely than women to overexpress HER2. Studies have shown that trastuzumab, a monoclonal antibody that targets HER2, dramatically improves outcomes in patients with HER2-positive MBC, who display aggressive clinical behavior [43]. Its function in promoting tumor invasion and metastasis through downstream activation of the Phosphatidylinositol 3-kinase (PI3K)/also called phosphoinositide 3-kinase (PI3K), and Mitogen-Activated Protein Kinase (MAPK) pathways has been highlighted by the observation of dysregulation of the epidermal growth factor receptor (EGFR), which is frequently linked to mutations or amplifications [44].

Mutations in tumor suppressor genes and oncogenes contribute to MBC pathogenesis. *BRCA2* mutations are the most frequently implicated mutations, accounting for up to 15% of MBC cases, with carriers facing an 8% lifetime risk of developing breast cancer [45]. *BRCA1* mutations, although less common, confer a more aggressive phenotype. *TP53* mutations associated with Li-Fraumeni syndrome have been identified in a subset of MBC patients and are correlated with early onset and poor prognosis [46]. Mutations in *CHEK2* and *PALB2* have also been linked to increased susceptibility to MBC, broadening its genetic landscape [13,47].

BRCA2 mutations are strongly associated with MBC, but the clinical effectiveness of Poly(ADP-ribose) polymerase (PARP) inhibitors in this population remains largely unknown. While PARP inhibitors like olaparib are FDA-approved for BRCA-mutated FBC and have shown promising results in *BRCA*-mutant prostate cancer, evidence in MBC is limited to anecdotal and small-scale reports [48–50]. A recent review underscores this gap, noting that, despite mechanistic rationale, “Evidence-based guidance for the treatment of MBC that have BRCA mutations is lacking” and most available data originate from retrospective series rather than controlled trials [50,51]. Given *BRCA2*’s role in homologous recombination repair, PARP inhibitors should theoretically induce synthetic lethality in MBC cells. However, without robust clinical trials focusing on male patients, this remains speculative. Consequently, treatment protocols for BRCA2-mutated MBC continue to be extrapolated from FBC or prostate cancer experiences, underscoring an urgent need for dedicated MBC clinical investigations.

Epigenetic modifications, including DNA methylation and histone acetylation, contribute to MBC progression by altering gene expression, without modifying the underlying DNA sequence. Hypermethylation of tumor suppressor gene promoters, such as *CDH1* and *RASSF1A*, leads to transcriptional silencing and unchecked tumor growth [52]. Histone modifications, particularly acetylation and methylation, play a pivotal role in chromatin remodeling, enabling the expression of oncogenes or the repression of tumor suppressor genes. These epigenetic alterations present opportunities for targeted therapies, such as histone deacetylase inhibitors, which are currently under investigation in clinical trials [53].

Noncoding RNAs, including microRNAs (miRNAs) and long noncoding RNAs (lncRNAs), are critical regulators of gene expression in MBC. miRNAs, such as *miR-21* and *miR-155*, act as oncogenes by targeting tumor suppressor pathways, whereas others, such as *miR-34a* and *miR-200c*, exhibit tumor-suppressive functions [54]. Dysregulation of lncRNAs, such as *HOTAIR* and *MALAT1*, has been implicated in promoting metastasis and chemoresistance, highlighting their potential as therapeutic targets [55]. The differential expression of these non-coding RNAs (ncRNAs) between MBC and FBC underscores the need for sex-specific molecular studies.

Comparative genomic and proteomic studies have revealed key differences between MBC and FBC. Although both share common pathways, such as ER signaling, the distinct molecular signatures in MBC reflect their unique biology. Proteomic analyses have identified the differential expression of proteins involved in immune regulation, apoptosis, and extracellular matrix remodeling, providing insights into potential therapeutic targets [56]. Genomic studies utilizing platforms such as The Cancer Genome Atlas (TCGA) have highlighted differences in mutational frequencies and copy number variations, emphasizing the heterogeneity of MBC [57].

NGS has revolutionized our understanding of MBC by enabling the comprehensive profiling of its genetic and epigenetic landscapes. Recent studies using whole-exome sequencing have identified novel mutations in genes such as *MAP3K1* and *GATA3*, which are involved in tumor progression and endocrine resistance [58]. Additionally, NGS has facilitated the identification of actionable mutations, paving the way for precise oncological approaches. For example, *PIK3CA* mutations present in approximately 20% of MBC cases that have been successfully targeted by *PI3K* inhibitors demonstrate the clinical utility of genomic insights [59].

**Figure 2:**
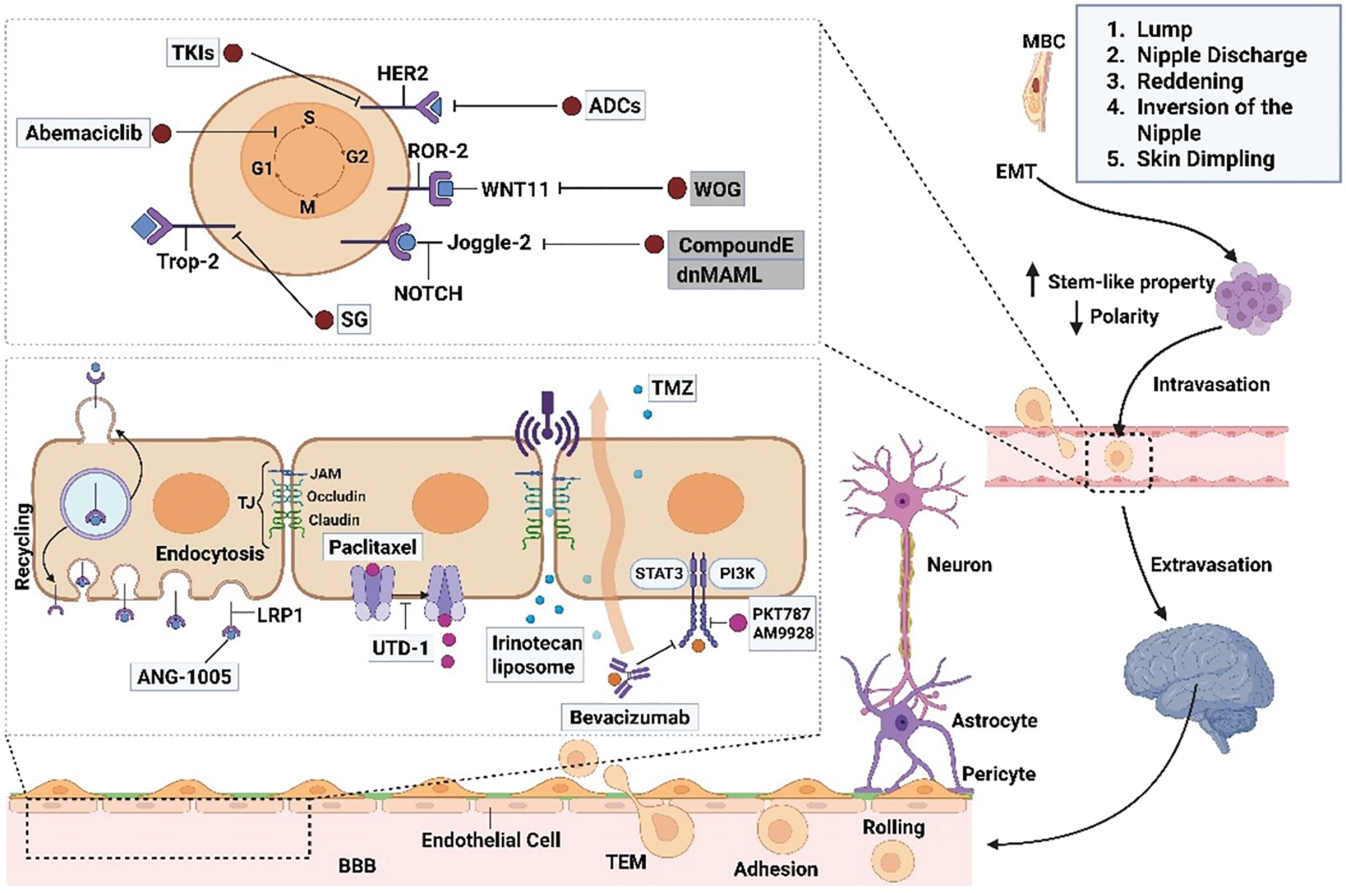
Breast cancer can metastasize to the brain through a complex sequence of cellular events. The process begins with epithelial-mesenchymal transition (EMT), which enables tumor cells to detach from the primary site and become invasive. These cells enter the bloodstream as circulating tumor cells (CTCs), aided by their interactions with macrophages and endothelial cells. To reach the brain, CTCs must traverse the blood-brain barrier (BBB) and navigate steps such as rolling, adhesion, and transendothelial migration (TEM). The BBB consists of endothelial cells, pericytes, astrocytes, and basement membranes and is typically sealed by tight junctions that restrict entry. However, CTCs can disrupt these junctions, increase permeability, and allow brain infiltration. Pericytes are vital for maintaining the BBB structure by supporting tight junctions and producing components of the basement membrane

The pathophysiology of MBC is significantly influenced by the tumor microenvironment (TME), which affects tumor growth, metastasis, and treatment resistance [60]. TME is made up of several biological components, such as immune cells, fibroblasts, endothelial cells, and extracellular matrix components. It interacts with tumor cells in a dynamic way to form a complex network that promotes the development of cancer [60]. Fig. 2 illustrates the intricate process by which MBC cells use several cellular and molecular routes to migrate to secondary sites for metastasis. Genetic mutations in BRCA1, BRCA2, and CHEK2 have been found to be prevalent in MBC, indicating different molecular pathways in its pathophysiology than in FBC. These genetic changes could affect the TME’s makeup and function, which could have an impact on the interactions between tumor cells and stromal and immune cells. MBC frequently manifests as a locally progressed disease with regional lymph node metastases due to the close proximity of male breast tissue to the skin. This suggests a potentially aggressive interaction between tumor cells and the surrounding milieu [61]. Developing tailored therapeutics requires an understanding of the distinct features of the TME in MBC. In order to disrupt these nexuses and maybe improve patient outcomes, new therapy approaches can be developed by figuring out how the TME interacts. Our understanding of the pathophysiology of MBC will be improved, and novel treatment targets will be found with the help of additional research into the TME’s function in the disease [62,63].

### 4.1 Recent Studies have Shed Light on the TME in MBC, Emphasizing Its Influence on Disease Progression and Potential Therapeutic Targets [61]

#### 4.1.1 Tumor-Infiltrating Lymphocytes (TILs) and Programmed Death-Ligand 1 (PD-L1) Expression

A subpopulation of tumors showing increased stromal TILs and PD-L1 expression was identified by a retrospective examination of 113 MBC cases. The inclusion of these characteristics implies that immune checkpoint inhibitor therapy may be beneficial for some MBC patients, even though the results were not statistically significant [61].

#### 4.1.2 Adipose-Inflammatory Microenvironment

Studies comparing the adipose tissue surrounding tumors in archival (1940–1970) and contemporary (1998–2006) MBC cohorts revealed significant changes. Crown-like structures (CLS), which are characterized by dead adipocytes encircled by macrophages, were more common in the current cohort, and CD8 and CD68 expression were elevated. The association between these inflammatory characteristics and worse survival underscores the part played by the adipose-inflammatory milieu in the pathophysiology of MBC [64].

#### 4.1.3 Single-Cell Transcriptomic Analysis

A study that compares TME from MBC and FBC using single-cell RNA sequencing. MBC showed increased fatty acid metabolism in this study, which was linked to immune infiltration attenuation and the spread of cancer cells. Furthermore, T-cells in the MBC microenvironment showed abnormal and unique metabolic processes, indicating possible treatment targets [65].

#### 4.1.4 Cancer-Associated Fibroblasts (CAFs)

CAFs are important elements of the TME and have been demonstrated to increase the malignancy of breast cancer by a number of processes, such as the release of nutrients, the production of exosomes, the secretion of factors (chemokines), the modification of the extracellular matrix, and the inhibition of immune cell activity. These interactions may affect the effectiveness of treatment and aid in the growth of tumors [66]. These results demonstrate how important the tumor microenvironment is in MBC, with elements including TILs, PD-L1 expression, and inflammation of adipose tissue influencing the course of the illness. Additionally, they highlight encouraging prospects for targeted medicines that aim to improve treatment results by modifying the microenvironment.

## 5 Current Therapeutic Strategies for MBC

### 5.1 Standard Treatment Modalities

The most common surgical procedure for MBC is mastectomy, which continues to be the mainstay of treatment. Male breast tissue has a limited volume; thus, mastectomy is frequently chosen over breast-conserving surgery (BCS), which is only practical in certain situations when sufficient margins may be obtained without sacrificing appearance [67]. Sentinel lymph node biopsy (SLNB) and axillary lymph node dissection (ALND) are frequently carried out for therapeutic and staging reasons. Recent research has demonstrated that, in carefully chosen patients, the oncological results after mastectomy and BCS are similar, highlighting the significance of customized surgical planning [68].

In MBC instances with big tumors, positive LNs, or invasion of the chest wall, radiation therapy is recommended. Radiation therapy following mastectomy improves overall survival and dramatically lowers local recurrence rates, especially in high-risk patients [69]. Radiation treatment advancements, including deep inspiration breath hold (DIBH) and intensity-modulated radiation therapy (IMRT), have decreased long-term toxicities, increased precision, and lowered exposure to adjacent normal tissues [70]. However, the development of optimal protocols for this population is limited by the lack of radiation research specifically focused on males. Management of MBC requires systemic medication, especially for HER2-positive and hormone receptor-positive subtypes. Tamoxifen is the recommended treatment for ER-positive and/or PR-positive MBC, and hormonal therapy is the cornerstone of this approach [43]. Although adherence may be hampered by side effects such as hot flashes and thromboembolic events, tamoxifen successfully lowers the risk of recurrence and increases survival in these patients. Although aromatase inhibitors are useful for postmenopausal women, they are not very effective for men unless they are used in conjunction with analogues of gonadotropin-releasing hormone (GnRH) to block the generation of testosterone in the testicles [71]. In aggressive or hormone receptor-negative instances, chemotherapy is used; the most common regimens are based on anthracyclines and taxanes. When BRCA mutations or basal-like characteristics are present, platinum medications may be taken into consideration [72]. The results of HER2-positive MBC have changed as a result of HER2-targeted treatments, such as trastuzumab and pertuzumab. Studies extrapolating from FBC trials showed that trastuzumab greatly increased overall survival (OS) and disease-free survival (DFS) when paired with chemotherapy. For advanced disease, dual HER2 inhibition using trastuzumab and pertuzumab is advised because it is more effective than single-agent regimens [73]. However, the dearth of information on HER2-positive illness that is particular to men highlights the necessity of involving MBC patients in clinical trials.

### 5.2 Challenges in Tailoring Treatment

One major issue is the underrepresentation of MBC patients in clinical trials, which forces the use of extrapolated data from FBC research [74]. The distinct biochemical and clinical subtleties of MBC may not be adequately captured by this extrapolation, which could lead to either overtreatment or undertreatment in some circumstances. Treatment adherence is another major issue; research shows that men are less likely than women to adhere to their treatment plans, frequently as a result of psychosocial or treatment-related toxicities [75]. Addressing these challenges requires the establishment of male-specific registries and clinical trials to generate robust evidence to tailor therapeutic strategies.

### 5.3 Emerging Therapies

Numerous *in vitro* and *in vivo* investigations have been carried out to evaluate the therapeutic potential of small compounds, chemotherapy, or antibiotics in MBC, or to find possible biomarkers. Table 1 provides a partial list. By preventing cell cycle progression, Cyclin-Dependent Kinase 4 and 6 (CDK4/6) inhibitors, such as palbociclib and ribociclib, have demonstrated potential for treating hormone receptor-positive MBC. Even though the majority of the data came from research on women, trials are currently being conducted to assess the safety and effectiveness of these drugs in men, and early findings point to similar results [76]. Similarly, PARP inhibitors, such as olaparib and talazoparib, have demonstrated efficacy in *BRCA*-mutated MBC by exploiting synthetic lethality, offering a targeted approach for patients with hereditary mutations [77].

**Table 1:** *In vivo* and *in vitro* studies in male breast cancer

*In vivo*/*In vitro* studies	Model	Outcomes	References
Effect of antineoplastic drugs in a male spontaneous mammary tumor model	*In vivo* (male PyVT transgenic mouse model)	Evaluated tamoxifen, paclitaxel, and cisplatin. Cisplatin significantly reduced tumor growth; tamoxifen and paclitaxel were less effective.	[74]
MicroRNA expression profiling in MBC	*In vitro* (MBC tissue samples)	Identified unique microRNA expression profiles in MBC compared to FBC, suggesting distinct molecular pathways.	[76]
Intratumoral ER levels and estrogen-responsive gene expression in MBC compared with FBC	*In vitro* analysis (4 male and 7 female patients)	These findings indicate that estradiol is locally synthesized in MBC tissue via aromatase activity. Variations in estrogen-induced gene expression, potentially influenced by differing ERα/ERβ profiles, may reflect distinct estrogen functions in male compared to FBC.	[78]
Immunophenotyping of MBC-experience at a tertiary care centre	*In vitro* (analysis of 42 MBC cases)	High estrogen receptor positivity (97.6%) and progesterone receptor positivity (83.3%) were observed, with molecular subtyping revealing 64.3% luminal A and 35.7% luminal B cases, aiding in the biological behavior of MBC.	[79]
Cancer progression in mice of different genders inoculated with triple-negative 4T1 breast cancer cells	*In vivo* (male and female BALB/c mice inoculated with 4T1 cells)	The histological and immunohistochemical traits of MBC in BALB/c mice closely resemble stage IV human breast cancer. This mouse model enables the exploration of MBC mechanisms and may guide therapies, especially for male triple-negative cases.	[80]
Revealing two distinct subtypes of MBC using Gene Expression Profiling further led to the identification of a novel prognostic biomarker, i.e., N-acetyltransferase 1 (NAT1)	*In vitro* (analysis of 66 MBC tissue samples)	Two MBC subgroups (luminal M1 and luminal M2) with distinction in tumor biology and outcomes. An upregulation of *NAT1* led to better survival, which denotes it as a prognostic biomarker.	[81]
MBC tumors were profiled using a tissue microarray (TMA), a six-marker panel, and the PAM50 signature to know molecular subtypes	*In vitro* (analysis of 67 MBC cases)	Findings of the study suggest that MBC is predominantly a luminal B genomic disease, with significant discrepancies between IHC and PAM50 subtyping, especially in HER2-overexpressing cases, suggesting that HER2-negative cases cannot be ignored when there is an option to treat using anti-HER2 therapies.	[82]
Survival benefit of tamoxifen in MBC: prospective cohort analysis	*In vivo* analysis (448 patients)	Tamoxifen treatment was associated with improved Disease-free survival for MBC patients.	[83]
Development and validation of a nomogram to predict survival for advanced MBC	*In vitro* analysis (280 patients)	Time-dependent ROC and decision curve analyses showed that the nomogram outperformed the traditional Tumor, Node, Metastasis (TNM) staging system in predicting outcomes for advanced MBC. This highlights the nomogram’s reliability and strength as a predictive tool.	[84]
MBC: invasive carcinoma: ER, PR, and HER-2 expression was analyzed	*In vitro* (analysis of 91 MBC cases)	ER(+)ive (96.7%), PR(+)ive (92.3%), HER2 overexpression (16%) of MBC cases; Five-year overall survival was 68.1%, with poorer outcomes linked to grade 3 tumors and Ki-67 > 20%.	[85]
MBC: A Study was conducted to assess the correlation of ER, PR, HER2, Ki-67, and TP53 with treatment outcome	*In vitro* (study of 65 MBC cases)	TP53 and HER2 expression were found to be the key prognostic indicators, whereas ER, PR, and Ki-67 had limited predictive value. No significant improvement was found post-adjuvant therapies, though HER2 was linked with poor survival.	[86]
MBC profiling using PAM50 and IHC in the Danish Cohort	*In vitro* (analysis of 37 MBC cases)	PAM50 subtyping revealed luminal B (*n* = 56%), luminal A (*n* = 39%), and basal-like tumors (*n* = 5%); with a significant association between PAM50 and IHC surrogate markers (*p* < 0.001); luminal B, as compared with luminal A (*p* < 0.02), showed worse overall survival.	[87]

Note: BABL, BALB/c strain of mice; NAT1, N-acetyltransferase 1; TMA, Tissue Microarray; PAM50, Prediction Analysis of Microarray 50; IHC, Immunohistochemistry; HER2, Human Epidermal Growth Factor Receptor 2; ROC, Receiver Operating Characteristic curve; TNM staging system, Tumor, Node, Metastasis staging system; ER, Estrogen Receptor; PR, Progesterone Receptor; Ki-67, Ki-67 antigen (cell proliferation marker); TP53, TP53 gene.

BRCA2 mutations play a significant role in DNA repair via homologous recombination, making tumors with these mutations particularly susceptible to the PARP inhibitor (PARPi) through synthetic lethality. In FBC, this mechanism has translated into clinical benefit most notably in the OlympiAD and OlympiA trials, demonstrating significantly longer progression-free and disease-free survival with olaparib in BRCA-mutated cases [50,88–90]. However, male-specific clinical evidence remains extremely limited. Only anecdotal case reports and small retrospective reviews exist in men. A *BRCA2*-mutated male patient with leptomeningeal metastasis achieved a complete response with Olaparib [91]. Several female case series have demonstrated central nervous system penetration and efficacy in BRCA2 tumors, suggesting mechanistic plausibility in males [92]. Despite these promising signals, the absence of randomized or prospective trials involving male patients means no definitive clinical guideline yet supports PARPi use in BRCA2-mutated MBC. Current practice often extrapolates from FBC and BRCA-mutant prostate cancer data [50,93]. While mechanistic understanding and limited case reports justify the biological rationale for PARPi use in *BRCA2*-mutated MBC, there is a critical lack of gender-specific clinical trial evidence. Dedicated studies are urgently needed to establish safety, efficacy, dosing, and long-term outcomes in this population.

Immunotherapy is an emerging frontier in MBC treatment, with checkpoint inhibitors such as pembrolizumab showing activity in tumors with high PD-L1 expression or microsatellite instability (MSI) [55]. Early phase studies have explored the combination of immunotherapy with chemotherapy or targeted agents to enhance anti-tumor responses. However, the role of immunotherapy in MBC remains under investigation, and further research is required to identify predictive biomarkers for patient selection.

Precision oncology has revolutionized the treatment landscape by enabling the identification of actionable mutations using NGS. In particular, targeting *PIK3CA* mutations with alpelisib has shown efficacy in hormone receptor-positive MBC, thereby providing a tailored therapeutic approach [59]. The potential for targeting androgen receptors (AR) in AR-positive MBC is also being explored, with novel agents, such as enzalutamide, demonstrating preliminary activity in early phase trials [42]. These advancements highlight the promise of precision medicine in addressing the heterogeneity of MBC.

### 5.4 Patient Management in Metastatic Settings

The management of metastatic MBC requires a multidisciplinary approach to balance disease control with quality of life. Hormonal therapy remains the first-line treatment for hormone receptor-positive metastatic disease, with the sequential use of targeted agents, such as CDK4/6 inhibitors, to delay the onset of resistance [69]. For *HER2*-positive metastatic disease, the combination of trastuzumab, pertuzumab, and docetaxel has emerged as the standard of care, with later-line options including trastuzumab-emtansine (T-DM1) [73]. Platinum-based chemotherapy or PARP inhibitors are considered for *BRCA*-mutated metastatic cases. Case reports have highlighted the potential for prolonged survival with individualized treatment plans incorporating local therapies, such as radiation or surgery, for oligometastatic disease [70].

AI has emerged as a transformative force in the diagnosis, prognosis, and management of MBC. Given the unique challenges associated with MBC, including delayed diagnosis, underrepresentation in clinical trials, and lack of tailored treatment protocols, AI offers promising avenues for improving patient outcomes.

## 6 Role of Artificial Intelligence in Male Breast Cancer

### 6.1 Applications of AI in Male Breast Cancer

#### 6.1.1 Imaging and Pathology Analysis

AI technologies are revolutionizing imaging and pathology in MBC. Advanced AI algorithms can analyze mammograms, Magnetic Resonance Imaging (MRI), Computed Tomography (CT), and pathology slides to detect malignancies with high accuracy. These tools improve diagnostic consistency and reduce inter-observer variability. In mammography, convolutional neural networks (CNNs) have shown promise in enhancing detection accuracy, even outperforming radiologists in some studies [93,94].

The AI algorithm developed by Ibex Medical Analytics for breast biopsy review has demonstrated exceptional diagnostic accuracy, achieving an area under the curve (AUC) of 0.99, sensitivity of 95.5%, and specificity of 93.6% for invasive carcinoma [95,96]. For ductal carcinoma *in situ* (DCIS), it showed an AUC of 0.98, sensitivity of 93.2%, and specificity of 93.8%, underscoring its robustness in histopathological interpretation (Table 2) [96–98].

**Table 2:** Applications of AI in male breast cancer diagnosis, prognosis, treatment, and management

Sn.	AI applications	Functionalities	Reference
1	RlapsRisk™ BC by OWKIN	To determine optimal treatment pathways for early ER+/HER2- breast cancer patients, demonstrating improved cumulative sensitivity in comparison to standard clinical scoring systems.	[99]
2	Visiopharm ER APP	Aids in determining ER positivity and negativity in breast cancer tumors by providing outputs like the percentage of positive tumor nuclei and Allred score.	[99]
3	HER2 IHC App by Visiopharm	Utilizes the HER2-CONNECT™ algorithm for automated analysis of HER2-stained breast cancer tissue sections, offering scores per ASCO/CAP guidelines.	[99]
4	Google’s AI algorithm	Enhances breast cancer detection in mammograms, reducing false positives and negatives, and is integrated into commercial mammography services.	[94,100]
5	Deep learning models for TNBC	Integrate multiomics data to improve accuracy in subtype classification and prognosis prediction for TNBC.	[101]
6	Radiomics in axillary LN staging	Employs AI-driven radiomics to improve staging accuracy, potentially replacing invasive procedures.	[102]
7	AI foundation model “Chief”	Detects multiple cancer types, assesses treatments, and predicts survival rates with high accuracy by analyzing tissue slide images.	[103]
8	Deep-breast-cancer-recurrence (BCR)-Auto	Predicts breast cancer recurrence risk from routine H&E-stained whole slide images using deep learning, offering a cost-effective alternative for recurrence risk assessment.	[104,105]
9	AI model for tumor progression prediction	Identifies breast tumor stages likely to progress to invasive cancer, aiding clinicians in assessing cancer stage and potentially reducing overtreatment.	[106]
10	AI-enhanced mammography interpretation	Assists radiologists in detecting breast cancer in mammograms, reducing false positives and negatives, and improving diagnostic accuracy.	[107–109]
11	AI tool for predicting treatment side-effects	Predicts side-effects in breast cancer patients post-treatment, such as lymphoedema, enabling personalized treatment planning to reduce adverse effects.	[110]
12	MammaPrint	A genomic test that assesses the activity of certain genes in early-stage breast cancer to predict the risk of cancer recurrence, aiding in treatment decisions.	[111]
13	SimBioSys TumorScope	Utilizes AI and biophysical simulations to create spatially resolved virtual replicas of individual tumors, aiding in personalized treatment planning by predicting responses to therapy.	[112]
14	CPath TILs	An AI-based computational pathology tool designed to predict which patients with ductal carcinoma *in situ* (DCIS) are at higher risk of disease progression and would benefit most from radiation therapy.	[113]
15	AI model for metastatic detection	An AI model to improve the detection of breast cancer metastasis, potentially reducing the need for invasive biopsies.	[114]

Note: Visiopharm ER APP, Visiopharm Estrogen Receptor Application; ASCO/CAP, American Society of Clinical Oncology/College of American Pathologists; TNBC, Triple-Negative Breast Cancer; LN, Lymph nodes; Deep-BCR-Auto, Deep-Breast-Cancer-Recurrence-Auto; H&E, Hematoxylin & Eosin stain.

Mammography-based AI systems, across 36 FBC studies, report a pooled AUC around 0.90, with a sensitivity of 0.83 and specificity of 0.84. Similarly, MRI-based AI algorithms demonstrate comparable performance, with an AUC of 0.90, sensitivity of 0.86, and specificity of 0.82 [115–117]. Additionally, an international AI model for digital breast tomosynthesis showed a standalone AUC of 0.93 and improved radiologist performance from 0.90 to 0.92 in AUC, increasing sensitivity from 85.4% to 87.7%. Multiple AI mammography systems in female populations report pooled sensitivities of 85%–96% and specificities of 90%–97%, varying by cohort and modality [118].

However, no diagnostic imaging AI models (mammography, MRI, or ultrasound) have been clinically validated specifically in male cohorts. There are no published diagnostic accuracy metrics for AI applied to male breast imaging. The existing data pertain exclusively to FBC or general breast imaging AI. Consequently, the use of these AI tools in male patients remains unvalidated, and clinicians must continue relying on standard imaging and reporting practices until male-specific studies become available. This significant gap highlights an important research opportunity for designing and conducting male-specific clinical validation studies in AI-driven MBC diagnosis.

AI also supports tumor classification by analyzing mammographic density, ultrasound echogenicity, and histological features. For example, deep learning models have successfully identified histological subtypes like invasive ductal carcinoma and invasive lobular carcinoma (Table 2) [119,120].

#### 6.1.2 Immunohistochemistry and Tumor Grading

AI facilitates automated interpretation of IHC slides, expediting the assessment of biomarkers such as ER, PR, and HER2. This automation supports faster and more accurate treatment decisions [121]. Additionally, AI assists in tumor grading by evaluating mitotic counts, nuclear atypia, and lymphocytic infiltration (Table 2) [122].

A systematic review and meta-analysis evaluating AI performance in IHC for breast cancer reported high diagnostic accuracy in distinguishing *HER2* expression, with sensitivity ranging from 0.78 to 0.99 and specificity between 0.92 and 0.98, contingent on *HER2* status [123]. Parallelly, AI-based tumor grading models analyzing features such as tubule formation, nuclear pleomorphism, and mitotic count have demonstrated robust efficacy; notably, a deep learning algorithm achieved an AUC of 0.91 for breast cancer grade classification [102,124,125]. Although these studies predominantly focus on FBC, the shared histopathological characteristics suggest translational potential for MBC. However, the rarity of MBC limits the availability of sufficiently large and annotated datasets, impeding the development and clinical validation of AI models specifically for this cohort. Therefore, targeted research is essential to generate MBC-specific datasets and validate AI tools in IHC evaluation and tumor grading. Such efforts will advance AI-assisted diagnostics tailored to MBC, improving accuracy, reproducibility, and clinical outcomes for this underrepresented patient population.

#### 6.1.3 Predictive Analytics and Personalized Risk Assessment

Beyond imaging, AI enables personalized medicine by predicting disease progression and treatment response. It incorporates clinical, genetic, and environmental data to create individualized risk profiles, enabling early intervention [105]. AI-driven predictive analytics for MBC are still in the early stages, primarily due to the rarity of the disease and the limited availability of dedicated datasets. Nonetheless, recent studies applying AI models to breast cancer risk stratification and diagnosis, including MBC cases, have demonstrated promising diagnostic accuracy. For example, a machine learning model trained on combined male and FBC cohorts achieved a diagnostic accuracy of 87% and an AUC of 0.89 in distinguishing malignant from benign breast lesions, encompassing MBC cases [126]. In personalized risk assessment, AI models that integrate clinical, genetic, and imaging data predicted cancer risk with an AUC of 0.85, indicating moderate to high discriminative power. Deep learning algorithms applied to histopathological images for tumor subtype classification and prognostic stratification in BC reported diagnostic accuracies between 82% and 90%, with sensitivity and specificity exceeding 80% [105,127–129]. Furthermore, AI-assisted evaluation of IHC markers such as *HER2* and hormone receptor status in MBC showed sensitivities ranging from 0.80 to 0.95 and specificities between 0.88 and 0.96, comparable to benchmarks established in FBC [123]. Despite these encouraging findings, the limited scale of MBC-specific datasets continues to hinder robust clinical validation. Therefore, ongoing efforts to curate larger, dedicated datasets and develop tailored AI algorithms are essential to enhance diagnostic accuracy and enable personalized risk stratification in MBC.

### 6.2 AI in Understanding Molecular Mechanisms

#### 6.2.1 Predictive Modeling

AI-driven predictive models use RNA sequencing and multiomics data to forecast tumor behavior and treatment responsiveness. These models help identify actionable mutations for targeted therapies such as immune checkpoint and tyrosine kinase inhibitors [130]. Clinical validation data on AI-based predictive modeling aimed specifically at elucidating molecular mechanisms in MBC remain limited, primarily due to the rarity of MBC and the lack of large, dedicated datasets. Nevertheless, recent studies incorporating breast cancer cohorts that include or extrapolate to MBC cases have reported promising AI performance metrics.

#### 6.2.2 Integration of Multiomics Data

A multiomics AI model integrating genomic, transcriptomic, and proteomic data achieved approximately 85% diagnostic accuracy and an AUC of 0.87 in predicting molecular subtypes relevant to BC progression and therapeutic response [105]. Moreover, AI frameworks employing network-based modeling of signaling pathways implicated in BC reported sensitivities and specificities exceeding 82%, effectively enabling molecular risk stratification. Prognostic AI models utilizing molecular signatures have further demonstrated predictive concordance indices (C-index) above 0.80, underscoring their utility in forecasting clinical outcomes specific to BC [131–133].

These findings collectively underscore the considerable potential of AI-driven predictive modeling in advancing our understanding of the molecular mechanisms underlying BC. However, they also highlight the urgent need for larger, MBC-specific molecular datasets and thorough clinical validation. To refine these models and confirm their clinical relevance, it is essential to conduct studies on MBC-specific cohorts. While the current results are promising, further validation in larger, well-defined MBC populations is crucial to establish the diagnostic accuracy and clinical applicability of AI-based multiomics integration in this patient group.

#### 6.2.3 Optimization of Treatment Strategies

AI models assess biomarkers like tumor mutational burden and PD-L1 expression to determine potential responses to immunotherapy. This helps avoid ineffective treatments and optimize resource allocation [134,135]. Clinical validation data on AI-driven optimization of treatment strategies for MBC remain limited, primarily due to the rarity of the disease and the scarcity of large, MBC-specific datasets. In the absence of a dedicated MBC database, data are extrapolated from broader breast cancer studies. Recent AI-driven treatment planning approaches, including cohorts with MBC patients, show promise in predicting therapeutic response. However, their clinical validation in MBC remains limited, necessitating larger, disease-specific datasets and prospective studies to confirm diagnostic accuracy and clinical utility.

### 6.3 AI in Therapeutic Advances

According to a comprehensive review, performance measures such as accuracy and AUC for 64 studies assessing AI-based models for predicting treatment outcomes in breast cancer were largely high. Most of this research was constrained, though, by their retrospective designs, lack of external validation, and scant data/code sharing. To support clinical integration, the research highlights the need for prospective model creation, thorough external validation, and open-source openness [136].

A meta-analysis study of machine learning (ML) techniques for survival prediction found that deep learning and hybrid models (ML + Deep learning [DL]) performed better, with an average validation accuracy of ~90% (AUC ≈ 0.90). However, 80% of the studies only used internal cross-validation, indicating that external validation is still a major obstacle to clinical acceptance [137].

An investigation conducted in 2019 showed how AI might be used to automate radiation planning and organ-at-risk (OAR) segmentation, increasing consistency, decreasing clinician effort, and allowing for spatially guided dosage customisation based on recurrence risk [138]. Supporting this, a UK-based report indicated that AI-generated contours for breast and nodal targets required only minor modifications in 67%–89% of cases. Implementation of AI-driven planning significantly reduced CT-to-plan approval time from 12 to 7 days and facilitated the application of advanced techniques such as deep inspiration breath-hold (DIBH) with volumetric-modulated arc therapy (VMAT) [136].

A systematic review covering 2013–2024 reported high accuracy (84%–98%) for deep learning-based HER2 image analysis, yet only ~12% of the models underwent external validation—indicating a gap between experimental efficacy and clinical applicability [139].

The growing relevance of AI in immunotherapy prediction is highlighted by recent studies. Immunotherapeutic methods are being personalized through the use of multimodal AI methodologies that integrate genomes, imaging, and tumor microenvironment biomarkers [140,141].

According to a study conducted in 2024, AI models that combined clinical and multiomics data using techniques like support vector machines, random forests, XGBoost, reinforcement learning, and transfer learning were able to predict treatment response and survival with high accuracy (AUC ~0.91; accuracy 90%–96%) [141].

Moreover, integrated multi-modal AI platforms that combine radiomics, genomics, and clinical parameters are showing significant potential in advancing precision oncology in breast cancer [140,142,143]. AI is also being investigated for its utility in guiding treatment de-escalation. Specifically, tools are being developed to evaluate pathological response after neoadjuvant systemic therapy and support clinical decisions regarding the omission or minimization of surgery and/or radiotherapy [144].

There is a notable lack of studies on AI in MBC, as there are no reliable papers that report AI models created or validated in male cohorts. Despite significant physical and biological differences in MBC, including reduced breast volume and different patterns of hormone receptor expression, the majority of current AI applications in breast cancer are generated from data from female patients. Because of these variations, sex-specific AI models must be created in order to guarantee precise diagnosis, risk assessment, and treatment planning. A major need in the area is highlighted by the existing dearth of male-specific AI technologies, which also offers a great chance for innovation to enhance results for this underserved patient population.

### 6.4 Challenges and Future Directions

#### 6.4.1 Data Limitations and Ethical Concerns

Despite its potential, AI in MBC faces challenges such as limited datasets, especially for rare conditions like MBC. Ethical issues, including patient data privacy and algorithmic transparency, remain significant hurdles [101,145].

#### 6.4.2 Cost and Infrastructure

The high cost of AI implementation, including computational resources and data storage, limits accessibility, particularly in low-resource settings.

#### 6.4.3 Need for Clinical Integration

For AI to be effective, collaboration between clinicians and data scientists is crucial. Training clinicians in AI use and developing explainable models will promote trust and integration into clinical workflows [101,145].

#### 6.4.4 Continuous Improvement and Validation

Future research should focus on the continuous refinement of AI models and standardized validation through randomized trials to ensure clinical applicability and generalizability [130,145].

### 6.5 Implications for Sentinel Lymph Node Biopsy (SLNB)

SLNB is standard in FBC but remains underutilized in MBC. A retrospective study involving 220 MBC patients revealed that SLNB has comparable outcomes to ALND in early-stage disease, offering a less invasive alternative. AI could further support decision-making in axillary staging [146].

Clinical validation data specifically assessing AI applications in SLNB for MBC remain scarce, largely due to the rarity of MBC and limited dedicated studies. However, valuable insights have emerged from breast cancer cohorts inclusive of MBC cases, where AI-assisted imaging and pathological analyses demonstrated promising diagnostic performance in SLNB-related evaluations.

AI models utilizing preoperative imaging modalities such as ultrasound and MRI achieved diagnostic accuracies ranging from 85% to 92%, with sensitivities and specificities frequently exceeding 80%, thereby enhancing non-invasive assessment of nodal metastasis pertinent to BC patients [147]. In pathology, deep learning algorithms applied to sentinel lymph node slides reported diagnostic accuracies near 88%, with sensitivities and specificities above 85% in detecting micrometastases and isolated tumor cells, facilitating precise SLNB evaluation [148]. Furthermore, machine learning frameworks integrating clinical, imaging, and molecular data yielded AUC values between 0.87 and 0.90 for predicting SLNB positivity, supporting personalized surgical decision-making in BC [149–151]. Despite these encouraging findings, robust clinical validation involving larger, MBC-specific cohorts is essential to substantiate the diagnostic accuracy and clinical utility of AI-assisted SLNB in managing MBC.

## 7 Conclusion and Clinical Implications

Although uncommon, MBC has unique biology and clinical characteristics that necessitate targeted research and clinical care. Hormonal imbalances, lifestyle variables, and *BRCA2* mutations are important risk factors that contribute to its etiology. ER, PR, HER2, androgen receptor pathways, as well as epigenetic changes that increase therapeutic possibilities, have been clarified by developments in molecular profiling. Innovative diagnostic techniques that improve disease characterization, therapy monitoring, and prognostication include liquid biopsy and integrative multiomics approaches. However, the lack of MBC patients in clinical trials hinders the creation of gender-specific, evidence-based guidance. As listed below, addressing these issues calls for focused and useful research techniques.

### 7.1 Development of Standardized MBC Multiomics Datasets

The development of significant, well-selected, and standardized multiomics datasets especially for MBC is a fundamental first step. To capture illness heterogeneity, these should combine proteomic, epigenomic, metabolomic, transcriptomic, and genomic data from various populations. Meta-analyses and cross-study comparisons will be made easier by standardizing data collection procedures and annotation standards. Clinical metadata, including treatment plans, results, and comorbidities, must be included [152]. AI model training and validation will be accelerated by publicly available MBC-specific resources, aiding in the identification of new biomarkers and treatment targets.

### 7.2 Establishment of AI Model Validation Protocols Tailored to MBC

Since the unique biology and rarity of MBC, AI algorithms need strict validation procedures created especially for this group. To evaluate generalizability, validation frameworks should incorporate both internal and external cohort testing that is stratified by ethnicity, stage, and molecular subtype. Beyond accuracy, performance measurements must also include clinical utility indices, sensitivity, specificity, and AUC. To guarantee fair implementation, transparency in AI model interpretability and bias assessment is essential. Standardized performance reporting rules and benchmark datasets for MBC will encourage clinical adoption and reproducibility.

### 7.3 Promotion of Interdisciplinary Collaborations Bridging Computational and Clinical Domains

Multidisciplinary networks involving oncologists, pathologists, radiologists, geneticists, data scientists, and bioinformaticians are essential for advanced MBC research. These collaborations promote translational research by facilitating the fusion of computational advancements with clinical insights. Working together can make it easier to create multi-modal AI models that combine clinical, molecular, imaging, and pathology data to improve diagnosis, prognosis, and treatment planning. Multicenter studies can be coordinated, resources can be shared, and consensus standards can be developed by interdisciplinary consortia.

### 7.4 Targeted Experimental Designs and Data Integration Frameworks

Prospective, longitudinal cohort studies that concentrate on treatment response, resistance mechanisms, and survival outcomes in MBC should be incorporated into future research. Dynamic molecular alterations and therapeutic vulnerabilities will be revealed by experimental designs that incorporate multiomics profiling at various time points, including pre-treatment, post-treatment, and recurrence. Heterogeneous data types can be synthesized while maintaining patient privacy using data integration frameworks that make use of cutting-edge AI techniques (such as federated learning and graph neural networks). Personalized risk assessment and adaptive therapy optimization will be made possible by these methods.

### 7.5 Enhancing MBC Representation in Clinical Trials and Registries

Initiatives to encourage male participation in breast cancer clinical trials and create MBC-specific registries that collect therapeutic, demographic, and genetic data in order to close the crucial information gap. International cooperation can standardize procedures and make data exchange easier between areas, taking into consideration regional and ethnic differences in MBC biology and results. Campaigns for patient advocacy and education are crucial to raising men’s knowledge and involvement.

### 7.6 Multidisciplinary Care Models Incorporating Emerging AI and Molecular Advances

Coordinated multidisciplinary teams of oncologists, surgeons, radiologists, genetic counselors, and psychosocial support specialists are necessary for comprehensive MBC care. Precision oncology for male patients will be made possible by the incorporation of multiomics insights and AI-driven diagnostics into clinical processes. In order to detect hereditary risks, genetic counseling is essential, and shared decision-making frameworks maximize the choice of medication. It is possible to enhance early detection, track treatment response, and customize therapy by incorporating cutting-edge technology like digital pathology, liquid biopsy, and predictive analytics into standard care.

Targeted efforts are needed to close existing data and methodological gaps in order to further MBC research. Creating extensive multiomics datasets tailored to MBC that incorporate genomic, transcriptomic, proteomic, and epigenomic information from well-characterized cohorts and capture the distinct biological variety of MBC is a top aim. Effective AI training will be supported by standardized data collection and annotation, which will guarantee comparability. Setting up stringent AI validation procedures that are adapted to the unique biology and rarity of MBC is equally crucial. To guarantee accuracy, generalizability, and therapeutic relevance, AI models must be tested across a range of demographics and biological subtypes. Open reporting and bias evaluations further promote clinical acceptance and trust. By fusing cutting-edge AI methods with clinical insights, interdisciplinary partnerships between bioinformaticians, computer scientists, and doctors will spur innovation. Through this partnership, models integrating imaging, pathology, molecular, and clinical data can be developed, improving the accuracy of diagnosis and individualized care. To monitor molecular dynamics and treatment response, novel experimental designs are required, such as multi-modal and longitudinal research. Personalized risk assessment and adaptive medicines are made possible by AI-powered data integration frameworks that can synthesize diverse information while protecting patient privacy. When combined, these targeted research approaches will close gaps and hasten the conversion of multiomics and AI developments into better diagnosis, prognosis, and treatment. In the end, they aim to improve individualized treatment and results for MBC patients, an extremely underprivileged population in oncology.

## Data Availability

Not applicable (This article does not involve data availability, and this section is not applicable).
